# Testicular Dislocation After Unstable Pelvic Ring Injury

**DOI:** 10.7759/cureus.13119

**Published:** 2021-02-04

**Authors:** Zachary Bernhard, Devon Myers, Braden J Passias, Benjamin C Taylor, Joaquin Castaneda

**Affiliations:** 1 Medical Education, West Virginia School of Osteopathic Medicine, Lewisburg, USA; 2 Orthopedic Surgery, OhioHealth, Columbus, USA

**Keywords:** pelvic ring injury, testicular dislocation, orthopedic trauma, genitourinary trauma

## Abstract

Reproductive and genitourinary complications following pelvic ring injuries have been described; however, testicular dislocation is rare and can cause significant morbidity if not managed appropriately. We describe a case of testicular dislocation after pelvic ring injury and outline the subsequent management and outcome, and seek to identify areas of improvement to ensure expedient and appropriate care in the setting of these injuries.

Our case describes a 29-year-old male who presented to a level-one trauma center following a motorcycle collision. An anteroposterior compression type II rotationally unstable pelvic ring was identified on imaging. He was hemodynamically unstable and computed tomography (CT) with angiography was ordered. Arterial extravasation was noted from the bilateral anterior internal iliac arteries, which were subsequently embolized by interventional radiology. However, no concomitant genitourinary injury was identified at the time of CT. After resuscitation, the pelvis was stabilized with an anterior symphyseal plate and bilateral sacroiliac screws. During the anterior pelvic approach, the patient’s dislocated testicle was surprisingly discovered inferior to the pubis. Urology was consulted intra-operatively, and the testicle was successfully relocated. At the final follow-up, the pelvic ring was healed without any noticeable urogenital complication.

While testicular dislocation has been reported in the setting of pelvic ring injury, a paucity of information exists regarding management, implications, and areas for improvement in the identification of these injuries. Therefore, in cases of pelvic ring injury with significant trauma, radiologists, traumatologists, and orthopedic surgeons should adopt a multi-disciplinary approach in diligently attempting to rule out testicular dislocation pre-operatively. Intra-operatively, examination under anesthesia and careful operative technique are important in preventing iatrogenic injury.

## Introduction

Injuries to the pelvic ring account for approximately 12% of admissions to trauma centers each year [1]. Significant reproductive and genitourinary complications have been associated with traumatic pelvic ring disruptions. One rare complication is that of testicular dislocation. Testicular dislocation in the setting of pelvic ring injury is most commonly the result of “fuel tank” or “straddle”-type injuries, for example, in motorcycle accidents [2-7]. With recent investigations citing as few as 60 total cases [8], there exists a paucity in the current literature regarding accurate and expedient diagnosis as well as management of these injuries. If left unattended or mishandled, these injuries may lead to impaired fertility, hypogonadism, or testicular necrosis [1,2,4,8]. We present a case of unilateral testicular dislocation with pelvic ring instability that was not diagnosed until the time of surgery. We discuss the techniques and outcomes associated with its subsequent management and steps that may be taken to prevent any delays in diagnosis. The patient was informed that data concerning his case would be submitted for publication and he provided consent.

## Case presentation

A 29-year-old male was involved in a motorcycle collision. He was immediately brought to a level-one trauma center for advanced trauma workup. Examination showed no gross deformity to the extremities or pelvis and no significant ecchymosis or edema of the pelvic region. Immediate assessment revealed no obvious concern for neurovascular injury.

**Figure 1:**
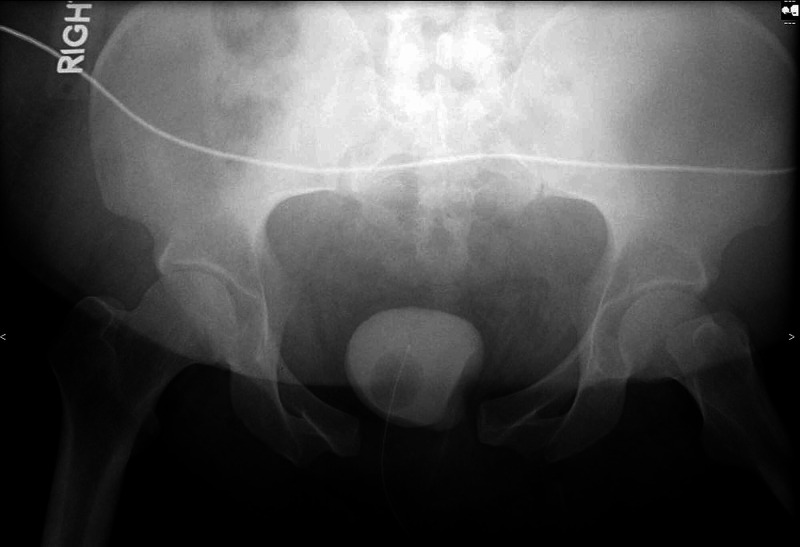
AP pelvis injury radiograph demonstrating APC type II pelvic ring injury. AP: anteroposterior; APC: anteroposterior compression

Soon after arrival, the patient began experiencing hemodynamic instability and received three units of packed red blood cells. Initial pelvic radiographs were not performed as the patient went straight to computed tomography (CT) per the trauma protocol at our institution. CT scans showed active arterial extravasation into the pelvis and he remained unstable, prompting immediate transfer to interventional radiology. Embolization of bilateral anterior internal iliac arteries, in combination with a resuscitative endovascular balloon occlusion of the aorta, was successful in halting bleeding and reestablishing hemodynamic stability. CT scans of the pelvis also demonstrated a pelvic ring disruption consistent with an anteroposterior compression (APC) type II injury (Figure 1).

**Figure 2:**
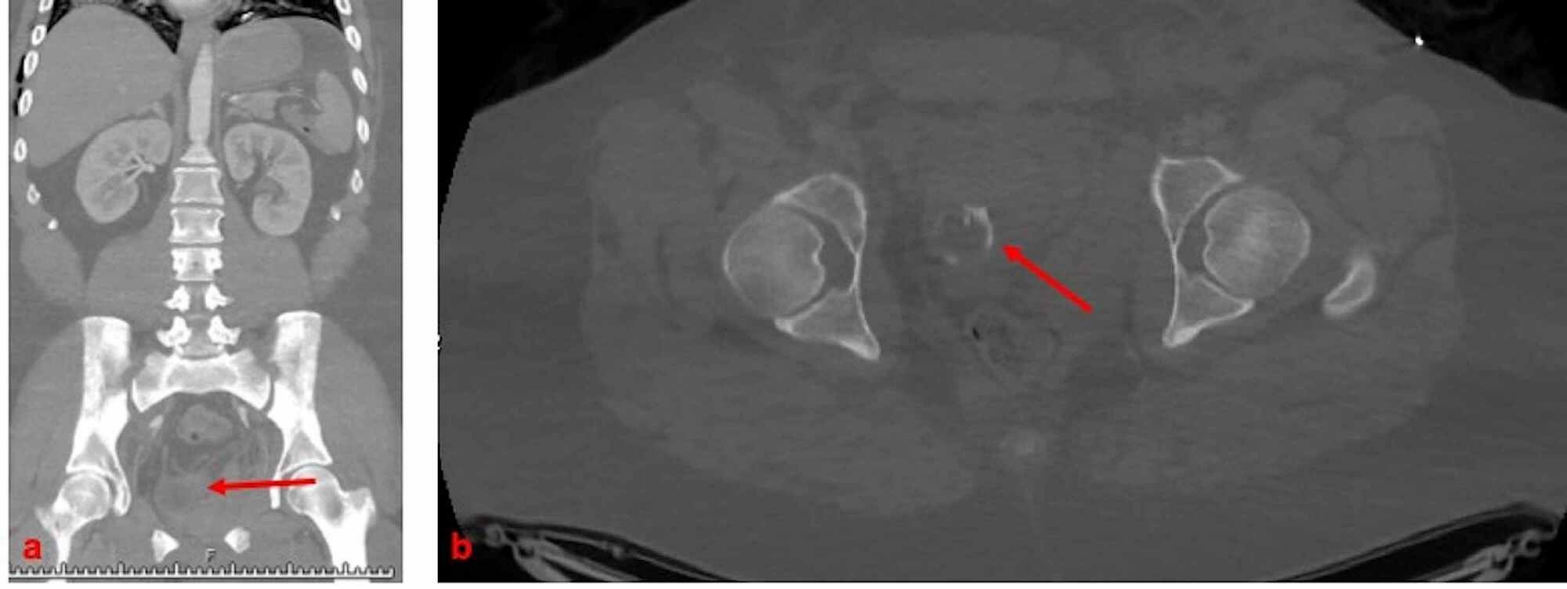
Preoperative CT scan demonstrating axial and coronal images of testicular dislocation adjacent to the pubis. CT: computed tomography

By the time the patient was available for orthopedic consultation, he was hemodynamically stable and additional pelvic radiographs were obtained. It was felt that as the reason for internal bleeding had been addressed and his hemodynamic parameters had stabilized, external fixation was not necessary. He was deemed fit for open reduction and internal fixation of his pelvis three days later after adequate resuscitation. Additionally, on retrospective evaluation of the CT scan, there was evidence of left testicular dislocation that was not read as such by the radiologist and not identified on exam (Figure 2). It was difficult to assess the viability of the testicle on CT.

**Figure 3:**
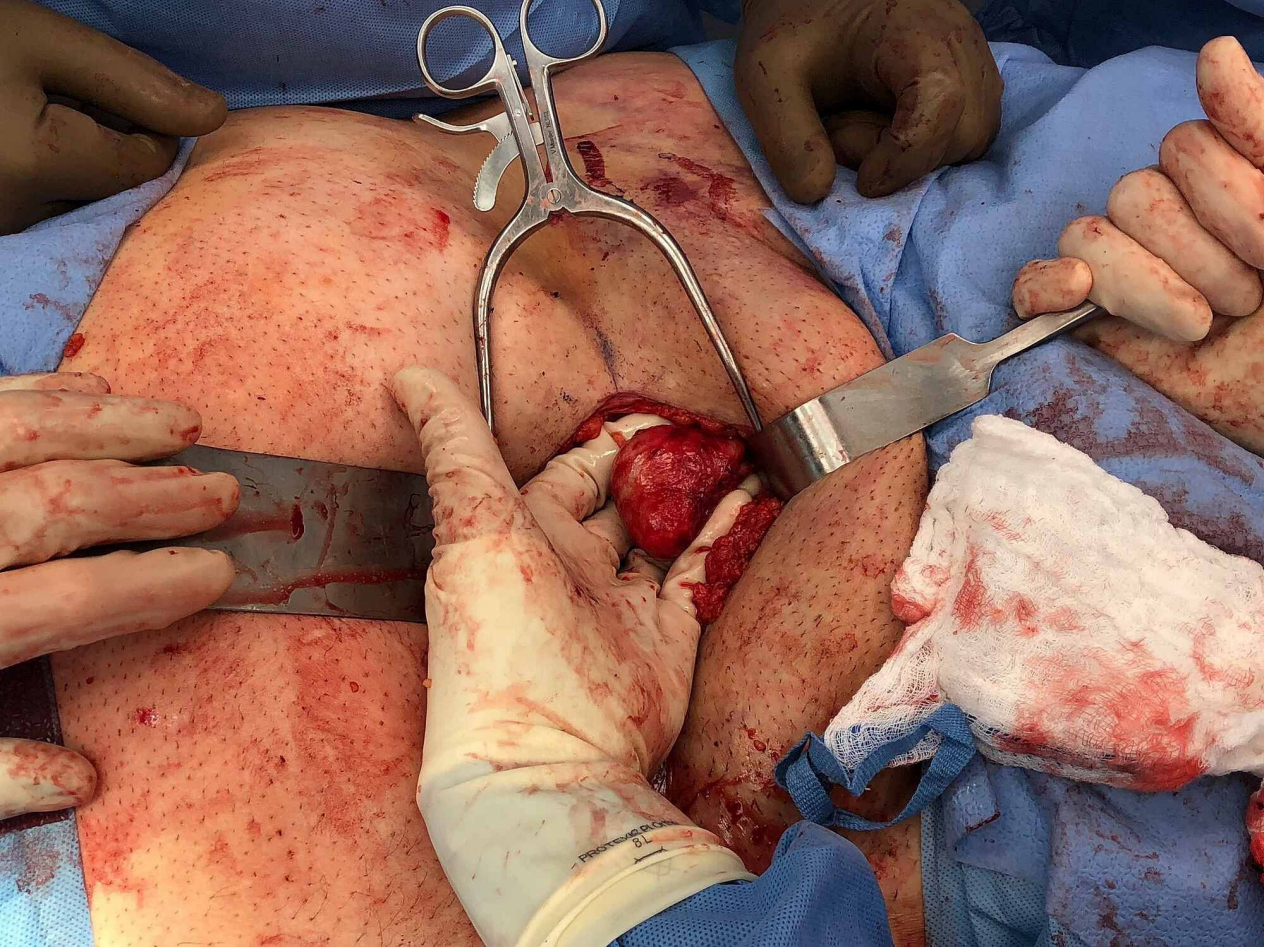
Intra-operative image demonstrating left testicular dislocation from beneath the pubic symphysis.

Next, the patient was brought to the operating room and placed supine on the table. Further clinical examination was performed under anesthesia, with no significant external edema, ecchymosis, scrotal injury, and open wounds noted. He was then prepped and draped in sterile fashion. The anterior pelvic ring was exposed utilizing a Pfannenstiel approach. After dissecting through a midline rectus split, significant diastasis of the pubic symphysis was clear. Disruption of the left rectus abdominus muscle insertion on the pubic tubercle was observed and remaining abdominal muscles were elevated off the pubic rami bilaterally. The patient’s left testicle was discovered extruding from the scrotum adjacent to the pubic symphysis (Figure 3).

This prompted immediate urology consultation. Appearing fully intact and highly mobile, the testicle was protected, and pelvic stabilization continued while awaiting urology. Anatomic reduction was achieved utilizing a Jungbluth clamp and two screws on either side of the pubic symphysis through a six-hole 3.5 mm pubic symphysis plate (Synthes; West Chester, PA, USA). After final alignment, all three screws on either side of the plate were filled.

**Figure 4:**
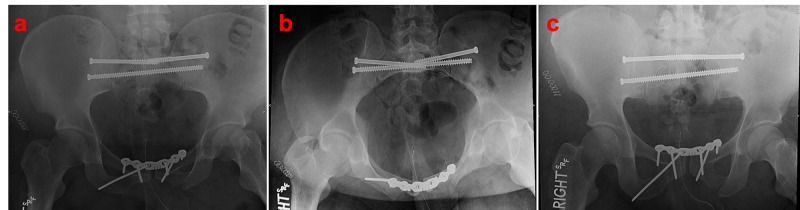
Postoperative AP (a), inlet (b), and outlet (c) radiographs demonstrating successful anterior and posterior pelvic ring fixation. AP: anteroposterior

Attention was turned to the bilateral sacroiliac joint widening. O-arm and Stealth navigation systems (Medtronic; Minneapolis, MN, USA) were utilized to achieve percutaneous stabilization. After guidewire positioning in both S1 and S2 sacral segments, three 7.3 mm cannulated screws were used to stabilize the posterior ring (Synthes; West Chester, PA, USA) (Figures 4).

After satisfactory reduction and stabilization was confirmed with fluoroscopy, urology successfully reduced the testicle to the hemiscrotum and secured it with vicryl suture. The urogenital structures were without compromise and the testicle and spermatocord appeared viable. The anterior wound was thoroughly irrigated, 2 g of vancomycin powder were placed deep in the pelvis, and the wound was closed in layers.

Following five weeks in a skilled nursing facility, the patient was discharged home with the assistance of home health and physical therapy. During his first follow-up visit, the patient reported continued pelvic pain, while denying urogenital complications including sexual dysfunction or difficulty voiding. Pelvic radiographs confirmed a well-aligned pelvic ring without evidence of hardware breakage or loosening. The decision was made at this point to progress his non-weight-bearing status to as-tolerated.

**Figure 5:**
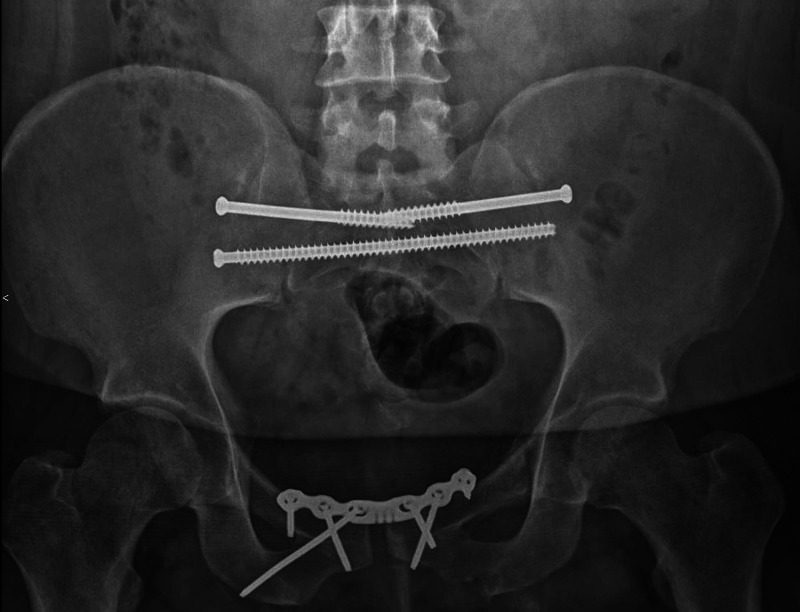
AP pelvis taken at final follow-up, demonstrating fracture union with maintenance of reduction and no evidence of hardware failure. AP: anteroposterior

The patient had his final follow-up at 13 months and was complaining of bilateral hip discomfort but minimal pain anteriorly or posteriorly in his pelvic ring. Repeat radiographic evaluation demonstrated fracture union with maintenance of reduction and no evidence of hardware failure (Figure 5).

Specific etiology of his bilateral hip pain was unclear at the time of follow-up but did not appear to be directly related to his pelvic ring fixation as this had healed uneventfully. He was likely significantly deconditioned and noted persistent psychosocial stressors which may explain some of these complaints. The scrotum and external genitalia remained intact at follow-up, without issue. He continued to deny sexual dysfunction or genitourinary complaints, suggesting that complications due to his associated testicular dislocation had been avoided.

## Discussion

Pelvic fractures are a notable cause of disability and chronic pain, and are the leading cause of morbidity and mortality in musculoskeletal trauma [9]. Our patient’s case supports the literature as he experienced significant polytrauma in addition to his pelvic ring disruption, resulting in persistent complaints of lower extremity pain. This is also consistent with studies suggesting worse clinical outcomes when a pelvic ring disruption is associated with genitourinary complications [9]. Bjurlin et al. published a retrospective study of 31,380 pelvic fracture patients, in which 1,444 patients had associated genitourinary injury (GUI). They concluded that cases with combined GUI and pelvic fractures resulted in longer hospital and intensive care unit stays, lower rates of discharge home, and increased mortality compared to pelvic ring fractures without GUI [9]. This study did not draw specific reasoning behind these correlations and highlights an importance for expanding the study of these injuries.

While GUI and other concomitant injuries are linked to higher morbidity and mortality, reasoning for such high mortality in these clinical situations is likely related to the severity of trauma and associated vascular disruption. Overall, 10-15% of patients with pelvic injuries and associated GUI arrive to the emergency department in hypotensive shock, and although not at arrival, our patient experienced early hemodynamic insufficiency [10,11]. While our patient survived, mortality associated with pelvic ring injuries ranges 32-45%, reiterating why early identification is imperative and expanding literature around early management of these injuries is beneficial [11-13]. Pelvic radiographs obtained at the time of a patient’s arrival in accordance with Advanced Trauma Life Support (ATLS) protocol provide important clues in addressing hemodynamic stability, and had this been performed immediately for our patient, a pelvic binder or sheet may have been placed to avoid initial hemodynamic decompensation while further imaging was performed. Fatal hemorrhaging is most commonly venous in nature from pre-sacral and lumbar venous plexuses or the fracture site itself [13-15]. In our case, arterial disruption was noted, which has been reported at a much lower frequency in the literature [13,14]. Though more rare, this scenario carries an even higher mortality risk, and swift embolization alongside ATLS measures are paramount to survival [11,13,15]. APC type II/III injuries are the most commonly reported injuries associated with arterial bleeding, and of these arterial sources, 67% come from the internal iliac artery [15]. Both scenarios were consistent in our case.

There are also several genitourinary and reproductive system implications in these cases that should not be neglected. The sexual complication most reported in pelvic ring injuries is erectile dysfunction, which is often secondary to pelvic fracture urethral injury (PFUI) [12,16-18]. This dysfunction is usually a side effect of shearing force in the bulbomembranous and prostatomembranous urethral junction interrupting cavernosal nerves [17]. In our patient, no gross deformity, edema, or ecchymosis of this region was noted on initial examination, nor did urology make note of any gross disruptions to the urogenital region that would have provided insight pre-operatively to this diagnosis. Fortunately, no sexual dysfunction was reported by our patient within the follow-up period.

Testicular dislocation is even more rarely encountered than injuries like PFUI. However, there are significant consequences if testicular dislocation is missed; therefore, it should always be closely assessed pre- and intra-operatively in pelvic ring injuries [1,2,4,8]. In the author’s opinion, this is the most important takeaway from this case. Radiologists are encouraged to closely evaluate for GUI injuries in the setting of a pelvic ring fracture. Additionally, the orthopedic surgeon must perform a thorough pre-operative examination and be aware of the possibility of testicular dislocation intra-operatively. The most common location to encounter a testicular dislocation is the superficial inguinal region, accounting for approximately 50% of the cases, while another 6% of the cases are truly abdominal such as in our patient [4]. In either instance, if dislocation is unbeknownst pre-operatively, there is inherent risk of iatrogenic urogenital injury in utilizing the Pfannenstiel incision [7]. While a Pfannenstiel approach was chosen in our case, the Stoppa approach has also been used in similar instances with variable outcomes [3,7,19]. Another complication of testicular dislocation is the suboptimal environment provided for fertility by the inguinal canal or abdomen. Hayami et al. provided some insight on this with a case of a four-month delayed testicular relocation. Impaired spermatogenesis was confirmed, persisting at six-month follow-up with slight improvement at eight months [20]. The conclusions of this study were two-fold; traumatic testicular dislocation negatively affects spermatogenesis, but timely relocation may allow for some recovery [20]. Provided no iatrogenic injuries occur to vital urologic structures, the outcomes of testicular dislocations alone are favorable, unlike the grim complications discussed with many GUI injuries [3]. Immediate recognition and reduction of mispositioned testicles is preferred, but delayed reduction may still provide viable testes and therefore should be attempted [4-6].

## Conclusions

In conclusion, attention should be paid to ruling out testicular dislocation in high-energy unstable pelvic ring disruptions, especially those requiring anterior surgical approach and stabilization. This combination of injuries not only poses a high initial mortality rate but is also associated with the potential for significant urologic morbidity, as outlined. While this patient fortunately survived and avoided iatrogenic urogenital injury, there are lessons to be learned from his management. Tightly adhering to ATLS protocol and scrutinizing all initial pelvic imaging while evaluating for potential concomitant testicular dislocation is critical. Although initial physical examination was reportedly unremarkable, this would be another place for emphasis. Swelling and ecchymosis in the scrotum and perineum should trigger detailed examination for any sign of testicular dislocation.

A paucity of literature exists on both the incidence and outcomes of management in such cases, but a multi-disciplinary approach is pivotal to maximizing outcomes in these patients. Our hope with this report is to expand the depth of literature on this topic while bringing attention to the existing literature. By sharing our experience and noting areas of oversight and improvement, we hope to contribute to the improved management of these injuries moving forward. In all, we report satisfactory pelvic alignment and fracture union, with no subsequent urologic complications at the final follow-up. Long-term follow-up and larger cohort review would be useful to further determine optimal management practices.
